# Massage Therapy in Children with Asthma: A Systematic Review and Meta-Analysis

**DOI:** 10.1155/2017/5620568

**Published:** 2017-05-21

**Authors:** Ji Wu, Xi-Wen Yang, Ming Zhang

**Affiliations:** ^1^Tian Shan Hospital of Traditional Chinese Medicine, Long Hua Hospital, Shanghai University of Traditional Chinese Medicine, Shanghai 200051, China; ^2^Shanghai Chest Hospital, Shanghai Jiaotong University, Shanghai 200030, China

## Abstract

**Objective:**

To systematically evaluate the efficacy of massage, a traditional treatment method of traditional Chinese medicine on children with asthma.

**Methods:**

Literatures from 5 databases using the date ranging from 1 January, 1990, to 13 December, 2016, were reviewed, which were all randomized controlled trials evaluating the efficacy on children with asthma and effect on lung function mainly by massage therapy.

**Results:**

14 researches with 1299 patients were included in the meta-analysis. Compared with control group, a better efficacy was found in treatment group, which focused on massage therapy. Compared with control group, there was remarkable increase on FEV1 as well as PEF in treatment group.

**Conclusion:**

All studies have shown that massage therapy has a significantly positive effect on children with asthma, improves the pulmonary function parameters of large airway, reduces the plasma concentrations of PAF and prostaglandin, and increases the levels of PAF-AH and DP1; therefore, it greatly improves pulmonary function. However, the limited research designs of included studies lead to high risk of bias. More randomized controlled trials with better methodological quality are needed to further confirm the effectiveness of massage.

## 1. Introduction

As a common chronic inflammatory disease of airways, asthma is characterized by multiple symptoms, tendency to relapse, reversible airway obstruction, and bronchial spasms [1]. The bronchoconstriction evoked by smooth muscle shortening promotes airway obstruction and constitutes the hallmark of asthma [2].* Asthma is a global health concern. It is the most common chronic disease of childhood. Although internationally there are wide variations in asthma prevalence, it affects over 300 million people worldwide [3, 4]. The World Health Survey estimates the global prevalence of asthma in adults to be 4.3% with as much as 21-fold variation among countries [5]*. It is estimated that the number of disability-adjusted life years (DALYs) lost due to asthma worldwide is about 15 million per year, which accounts for around 1% of all DALYs lost. The number of DALYs lost due to asthma reflects the high prevalence and severity of asthma, which is equal to that of diabetes, cirrhosis of the liver, or schizophrenia.* Underdiagnosis, undertreatment, exposure to air pollution, and unhygienic living conditions may contribute to a higher frequency and severity of symptoms of asthma in low-income communities [6]*. The financial burden on patients with asthma in different western countries ranges from $300 to $1,300 per patient per year [7], in view of the long duration and recurrent attacks, huge population, and the ever-increasing prevalence, the disease imposes a significant economic burden on the patient family and the whole society [8]. For example, the total cost ranges from £ 509 to 2281 per year for a single asthmatic patient [9]. These epidemiological data and economic burden survey* suggest* that it is urgent to have new methods and therapies in the clinic in order to prevent and control the asthma.

The immune response of asthma is typically associated with the expression of the Th2-type cytokines, such as interleukin- (IL-) 4, IL-5, IL-9, and IL-13 [10]. However, the understanding of the Th2-biased immune system in allergic asthma remains rudimentary and there are still no new immunomodulatory therapies to cure asthma [11]. In the clinic, *β*_2_-agonists and oral corticosteroids are still the first-line drugs for treating acute symptoms of asthma despite the presence of adverse effects [12]. The association between the use of *β*_2_-agonists and the risk of death has been frequently reported [13]. Corticosteroids cause detrimental side effects including immunosuppression and the increased risk of respiratory infections such as yeast infections in thrush [14].

In China, there are a number of traditional therapies for asthma [15], including acupuncture, herb medicine, and massage. Acupuncture is associated with the regulation of Th2 immunity and it is one of the most commonly used methods. Studies have showed that acupuncture could significantly reduce the nasal and saliva secretions of sIgA content in patient of allergic asthma. However, plasma cortisol levels showed no obvious statistical change after acupuncture treatment, which may indicate that antiasthmatic effect* proceeds* independent of glucocorticoid [16]. Massage has also been considered as a complementary treatment of asthma. Some report demonstrated that younger children who received massage therapy showed an immediate (30 min. after intervention) decrease in behavioral anxiety and cortisol levels [17]. Massage may enhance excitability of vagus nerve and reduce the cortisol level through partial pressure to the body, swinging, and vibration [18]. In addition, some researches also indicate that massage can obviously improve pulmonary function index of asthma patients [19]. However, there are very limited reports about massage therapy in children asthma, and therapeutic mechanism remains unclear [20].

Therefore, we utilized the existing data evaluating the therapeutic effect of massage on children with asthma. This review is a meta-analysis on the influence of this traditional technology on efficacy and pulmonary function index of children with asthma.

## 2. Methods

### 2.1. Retrieval Strategy

We have retrieved the following databases: PubMed, CNKI, Wanfang Database, CBM, and VIP. English databases were retrieved through “asthma” AND “massage OR manipulation OR an mo” and Chinese databases through “xiao chuan” AND “tui na OR an mo”. Publication dates ranged from January 1, 1990, to December 13, 2016. Languages were not limited. Only randomized controlled trials are included; animal researches, reviews, repeatedly published data, case-reports, and researches* lacking* the full text or data or incorrect researches were excluded.

### 2.2. Study Selection

All researches* meeting* the following criteria were included in the review: (1) design: randomized controlled trials (RCTs) were included; (2) patients: all participants in the study were children between 6 months to 15 years old and diagnosed with asthma; (3) interventions: treatment groups mainly focused on massage therapy combined with other basic therapies; control groups applied other basic therapies* besides* massage; (4) outcome: overall efficacy and results of pulmonary function tests (FEV1 and PEF) in treatment groups and control groups were collected; (5) included RCTs must include the index and data that this meta-analysis required.

### 2.3. Data Extraction

Two evaluators independently extracted data according to standards set in advance. If the related information had not been reported, we contacted the original author. First author and publication year were extracted as common information. The sample number, intervention, treatment duration, and the observation index were used to analyze the research characteristic. If there was any dispute, it was solved through discussion.

### 2.4. Risk of Bias

This study assessed the risk of bias according to the standards recommended by Cochrane handbook for systematic reviews of interventions (version 5.1) and used review manager software (version 5.1, the Nordic Cochrane Center, the Cochrane Collaboration, Copenhagen, Denmark): (1) random sequence generation; (2) allocation concealment; (3) blinding of participants and personnel; (4) blinding of outcome assessment; (5) incomplete outcome data; (6) selective reporting; and (7) other bias. According to this criterion, each study was rated as high risk of bias, low risk of bias, or unclear risk of bias. Necessary information was added through contacting with the authors. Any dispute was solved by discussion.

### 2.5. Data Synthesis and Analysis

STATA 12.0 (StataCorp LP, College Station, TX, USA) was used in this meta-analysis. For dichotomous data, the Risk Ratio (RR) and corresponding 95% Confidence Interval (CI) were calculated by Mantel-Haenszel method. For continuous variables, Standardized Mean Difference (SMD) and 95% Confidence Interval (CI) were applied. Heterogeneity between the studies was measured by *I*^2^; when *I*^2^ ≤ 50%, a fixed-effect model was used; when *I*^2^ > 50%, a random effects model instead.

## 3. Results

### 3.1. Characteristics of Studies

**Figure 1:**
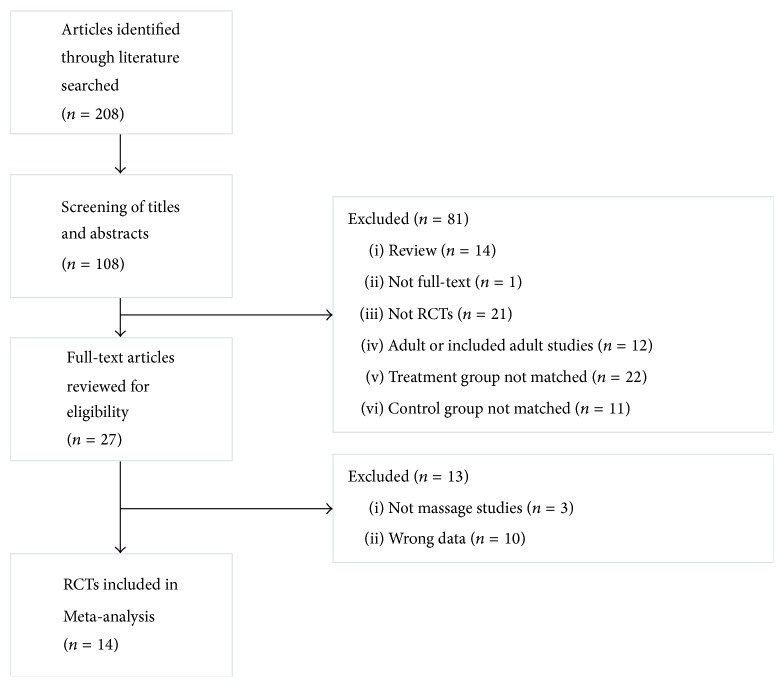
Flow diagram of study selection and identification. RCTs, randomized controlled trials.

14 eligible studies were included in this meta-analysis [21–34].  Figure 1 describes the selection process (literature identification and reasons for inclusion and exclusion). Studies with 1299 patients were included (range: 58–153) in our review.  Table 1 shows all characteristics of included studies.

### 3.2. Bias Risk Assessment

**Figure 2:**
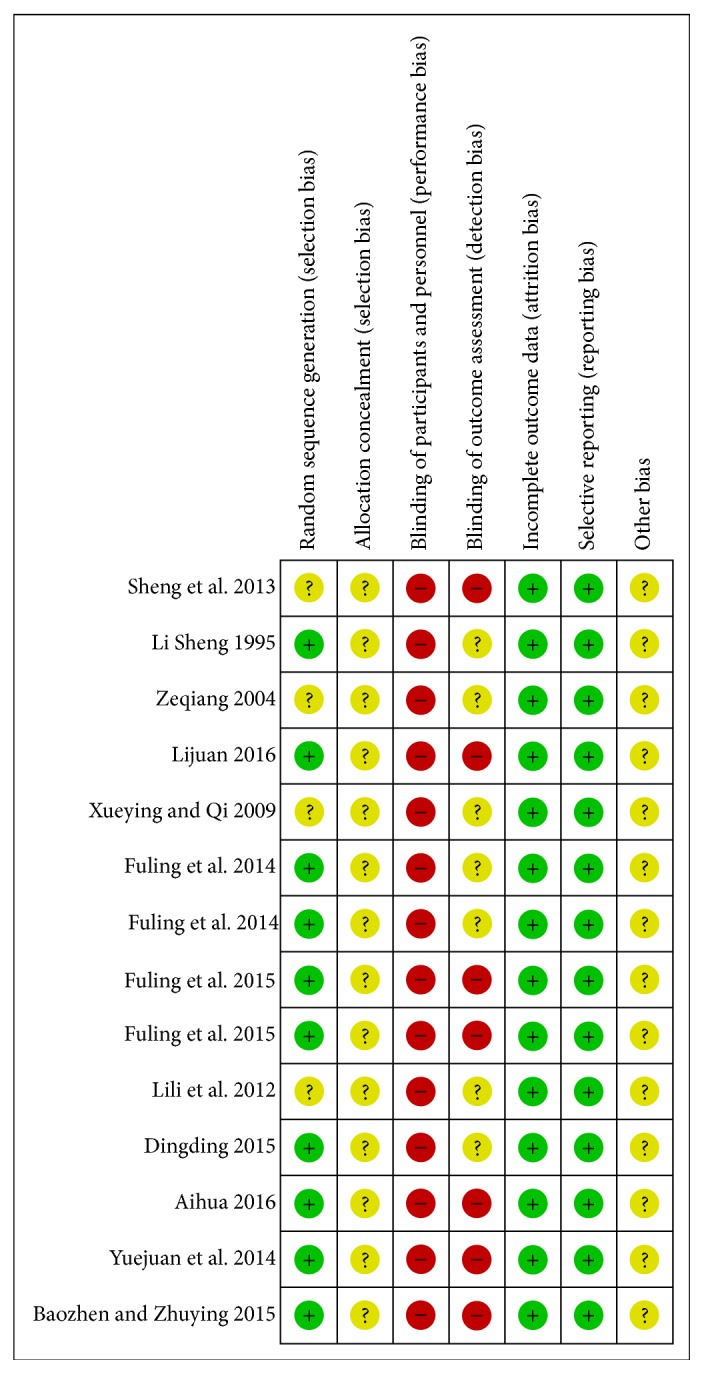
Risk of bias. Every domain was classified as “low risk of bias,” “high risk of bias,” or “unclear risk of bias.”

Figure 2 shows the risk of bias in the included studies. For the generation of random sequences, 10 studies were considered to be of low risk of bias [21, 22, 24, 26–30, 33, 34]. In terms of concealment random allocation,* all* studies* included* were considered to be of unclear risk of bias [21–34]. In terms of blinding method of participants and implementers, all studies were classified as high risk of bias. For blind method of results appraisal, 7 researches were considered to be* at* unclear risk of bias [23, 26, 27, 30–33], but other 7 were classified* as* the group of high risk of bias [21, 22, 24, 25, 28, 29, 34]. For incomplete data results and selective reports, all studies* included* were of low risk of bias. All studies* included* were considered to be of unclear risk of bias on “other bias” item [21–34].

### 3.3. Percentage of Total Efficacy

**Figure 3:**
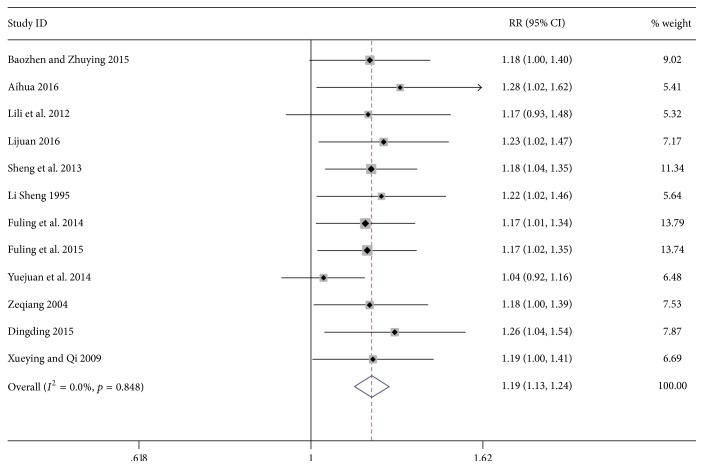
Forest plot showing the effect of massage application for children asthma on overall efficacy.

12 studies including 1099 patients [21–32] compared the efficacy of basic treatments combined with or without massage treatment on children with asthma. The comprehensive results* showed* a significantly higher efficacy in the massage group than the control group (RR 1.19; 95% CI 1.13–1.24; *p* = 0.001; *I*^2^ = 0%, Figure 3).

### 3.4. Pulmonary Function Testing

**Figure 4:**
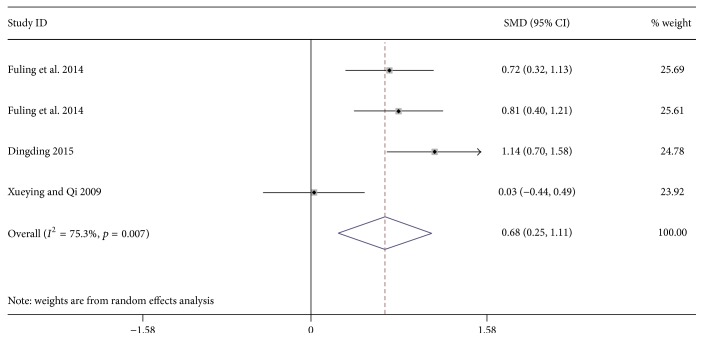
Forest plot showing the effect of massage application for children asthma on FEV1. FEV1 = forced expiratory volume in 1 second.

**Figure 5:**
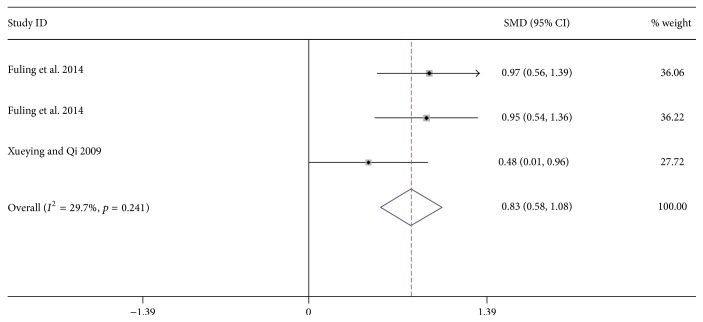
Forest plot showing the effect of massage application for children asthma on PEF. PEF = peak expiratory flow.

4 researches including 365 patients reported the influence of massage on forced expiratory volume in 1 second (FEV1) on children with asthma [31–34]. In addition, 3 studies with 271 patients reported the effect of massage on maximal expiratory flow (PEF) [32–34]. Results show that massage-focused therapy can significantly improve FEV1 (SMD: 0.68; 95% CI: 0.25 to 1.11; *p* = 0.002; *I*^2^ = 75.3%, Figure 4) and Peak Expiratory Flow (PEF) (SMD: 0.83; 95% CI: 0.58 to 1.08; *p* = 0.002; *p* = 0.001; *I*^2^ = 29.7%, Figure 5).

## 4. Discussion

In this meta-analysis, we evaluated the effects of massage on treatment of children asthma. The findings produced by the fixed-effects model* indicate* that massage therapy can significantly increase the efficacy of the treatment on children asthma and improve pulmonary function index FEV1 and PEF. However, we could not conclude the favorable mechanism of massage in the overall meta-analysis due to the small number of articles.

As far as we know, one of the disadvantages of case-control study is information bias. We also cannot ignore the role of confounding bias without information bias. Moreover, the symptoms of asthma maybe relieved, after children became adults. Therefore, we conducted a meta-analysis that included studies adjusting the interference of these factors. 14 researches with 1299 patients were included in the meta-analysis. Compared with control group, a better efficacy was found in treatment group, which focused on massage therapy (Risk Ratio [RR], 1.19; 95% Confidence Interval [CI], 1.13–1.24; *p* = 0.001; *I*^2^ = 0.0%).

This systematic review aims to evaluate the effects of massage on treatment of children asthma. According to the traditional Chinese medicine (TCM), a sound healthy status can be described as the balance of body Qi and blood, Yin and Yang. Pathogenesis of TCM focuses on the strength of the body Qi and indicates that the key factors responsible for diseases lie in the deficiency of Qi, the disorder of Qi ascending and descending movement, and stagnancy of blood. Clinically, weakened protective-energy is induced by deficiency of vital energy, and humans with Qi-deficiency are more likely to be invaded by exogenous pathogen, which leads to the pathological changes of internal organs and meridians. In addition, ancient Chinese physicians believed that retention of phlegm and fluid was an internal cause of recurrent asthma attacks. They concluded that the formation of phlegm resulted from the disturbance of Qi-movement, water absorption, and microscopic transfusion. Thus, the central therapeutic target of massage is to remove Qi stagnation, which helps to reduce associated pathogenesis of bronchial asthma. Previous researchers found that massage can improve the pulmonary function and immunity of children with asthma [33, 34]. In the research of Fuling et al., they enrolled 100 patients to investigate* into* the effect of massage on lung function of pediatric asthma in the acute phase [33]. Their data showed that compared with the control group, pulmonary function parameters of large airway (FVC, FEV1, and PEF) were improved noticeably in the treatment group with traditional techniques of pediatric massage. However, the parameters of small airways function (FEF25, FEF50, and FEF75) were not ameliorated after massage therapy, indicating the mechanism of massage in pulmonary function regulation. In another trial performed by Fuling et al., they revealed that pediatric massage could effectively reduce the plasma concentrations of PAF and prostaglandin and increase the levels of PAF-AH and DP1,* accompanied* by a significant improvement in the clinical symptoms of childhood asthma [34]. In addition, massage therapy can bring a series of benefits to parents and children: the treatment is free of charge and can be* conducted* at home by parents or guardians. There is almost no side effect as long as* it is in* correct operation. Regular massage therapy can also reduce the cost of medication and improve the quality of life of children and their families [19]. However, there is no clear evidence-based medical support about the value of massage in improving efficacy of asthma treatment as well as lung function in children. This meta-analysis found that massage therapy can significantly increase the efficacy of the treatment on children asthma and improve pulmonary function index FEV1 and PEF. In the process of diagnosis and treatment for children with bronchial asthma, pulmonary function measurement is a very important means and provides a reliable basis for guiding treatment, monitoring condition, evaluating curative effect, and judging prognosis, so that children with asthma can be treated* by* systemic standard [35]. In addition, FEV1 and PEF reflect the degree of large airway resistance and are used to classify asthma severity in children [19, 33]. Therefore, our study shows that the severity of asthma in children will decline eventually if patients* have* received massage treatment.

Several limitations should also be detailed when* it comes to* interpreting the results in this meta-analysis: (a) only a total of 14 randomized controlled trials were included in this review, in which the control groups were different. (b) The treatments were carried out in conjunction with other basic therapies, and the area, frequency, and duration of massage were different* from* each other, so we were unable to remove potential confounding factors. (c) Most studies did not include the change of FEV1 and PEF, resulting in a small number of studies* included*. (d) The study subjects mainly came from China; these may affect the results of external validity.

## 5. Conclusion

In the meta-analysis, our results* show* that massage therapy can effectively treat asthma and significantly improve pulmonary function in children.* Considering* the poor methodological quality, small samples, and lack of follow-up data of included studies, more randomized controlled trials of multicenter, large sample and enough follow-up duration are needed to better confirm current findings.

## Figures and Tables

**Table 1:** Characteristics of the included studies.

Included literatures	Sample number (treatment group/control group)	Interventions		Treatment duration	Observation index
		Treatment group	Control group		
Baozhen and Zhuying 2015 [21]	50/50	Massage + conventional spray treatment (Beclomethasone Dipropionate Aerosol)	Conventional spray treatment (Beclomethasone Dipropionate Aerosol)	8 weeks	Total efficacy (%)

Aihua 2016 [22]	34/30	Massage + Chinese medicine application + conventional nursing	Conventional nursing	28 days	Total efficacy (%)

Lili et al. 2012 [23]	30/30	Massage + psychological intervention + Glucocorticoid inhalation	Glucocorticoid inhalation	6 months	Total efficacy (%)

Lijuan 2016 [24]	40/40	Massage + Salmeterol and Fluticasone powder inhalation	Salmeterol and Fluticasone powder inhalation	12 weeks	Total efficacy (%)

Sheng et al., 2013 [25]	60/60	Massage + acupoint catgut-embedding	Montelukast sodium tablets	3 months	Total efficacy (%)

Li Sheng 1995 [26]	20/38	Massage + Dingchuan decoction	Dingchuan decoction	15 days	Total efficacy (%)

Fuling et al. 2015 [27]	76/77	Massage + Beclomethasone Dipropionate Aerosol	Beclomethasone Dipropionate Aerosol	3 months	Total efficacy (%)

Fuling et al. 2015 [28]	77/76	Massage + Beclomethasone Dipropionate Aerosol	Beclomethasone Dipropionate Aerosol	3 months	Total efficacy (%)

Yuejuan et al. 2014 [29]	30/30	Massage + Supermicro Dingchuan decoction	Pulmicort Respules + Ventolin inhalation	10 days	Total efficacy (%)

Zeqiang 2004 [30]	56/30	Massage + acupuncture	Aminophylline + Prednisone	10 days	Total efficacy (%)

Dingding 2015 [31]	47/47	Acupoints massage + Zhisou decoction and Ephedrine powder	Montelukast sodium tablets	15 days	Total efficacy, FEV1

Xueying and Qi 2009 [32]	38/33	Foot acupoints massage + hot-water soak	Hot-water soak	6 months	Total efficacy, FEV1

Fuling et al. 2014 [33]	50/50	Massage + spray therapy (Ventolin + Budesonide)	Spray therapy (Ventolin + Budesonide)	—	FEV1, PEF

Fuling et al. 2014 [34]	50/50	Massage + BDP	BDP	3 months	FEV1, PEF

FEV1 = forced expiratory volume in 1 second; FVC = forced vital capacity.
